# Frequency of Children Diagnosed with Perinatal Hepatitis C, United States, 2018–2020

**DOI:** 10.3201/eid3001.230315

**Published:** 2024-01

**Authors:** Suzanne M. Newton, Kate R. Woodworth, Daniel Chang, Lindsey Sizemore, Heather Wingate, Leah Pinckney, Anthony Osinski, Lauren Orkis, Bethany D. Reynolds, Cynthia Carpentieri, Umme-Aiman Halai, Caleb Lyu, Nicole Longcore, Nadia Thomas, Aprielle Wills, Amanda Akosa, Emily O’Malley Olsen, Lakshmi Panagiotakopoulos, Nicola D. Thompson, Suzanne M. Gilboa, Van T. Tong

**Affiliations:** Centers for Disease Control and Prevention, Atlanta, Georgia, USA (S.M. Newton, K.R. Woodworth, E.O. Olsen, L. Panagiotakopoulos, N.D. Thompson, S.M. Gilboa, V.T. Tong);; Eagle Global Scientific, LLC, San Antonio, Texas, USA (D. Chang, A. Akosa);; Tennessee Department of Health, Nashville, Tennessee, USA (L. Sizemore, H. Wingate);; Massachusetts Department of Public Health, Boston, Massachusetts, USA (L. Pinckney, A. Osinski);; Pennsylvania Department of Health, Pittsburgh, Pennsylvania, USA (L. Orkis, B.D. Reynolds);; Chickasaw Nation Industries, Norman, Oklahoma, USA (C. Carpentieri);; Los Angeles County Department of Health, Los Angeles, California, USA (U-A. Halai, C. Lyu);; New York State Department of Health, Albany, New York, USA (N. Longcore, N. Thomas);; New York City Department of Health and Mental Hygiene, New York City, New York (A. Wills)

**Keywords:** Hepatitis C, children, perinatal, viruses, United States

## Abstract

We describe hepatitis C testing of 47 (2%) of 2,266 children diagnosed with perinatal hepatitis C who were exposed during 2018–2020 in 7 jurisdictions in the United States. Expected frequency of perinatal transmission is 5.8%, indicating only one third of the cases in this cohort were reported to public health authorities.

Hepatitis C virus (HCV) can be transmitted perinatally (*1*). Rates of acute HCV infection have increased recently (*2*), but few children perinatally exposed to HCV are tested and referred to care (*3*). As of November 2023, the Centers for Disease Control and Prevention recommends testing of all perinatally exposed infants for detection of HCV RNA at age 2–6 months, which is earlier than previous recommendations of ≥18 months of age for HCV antibody testing (*4*). There may be advantages to performing HCV RNA testing earlier, before children might become lost to follow-up (*5*). A prior analysis found only 16% of children perinatally exposed to hepatitis C in Philadelphia, Pennsylvania, USA, received HCV testing (*6*). Limited data are available from larger surveillance cohorts about current testing patterns of children perinatally exposed to HCV.

Positive HCV test results are nationally notifiable in the United States, but negative HCV test results are not. To identify potential gaps in testing and surveillance, we used positive HCV test results to describe testing and frequency of children diagnosed with perinatal hepatitis C during 2018–2020 compared with the expected frequency of perinatal transmission in 7 US jurisdictions. This activity was deemed as public health surveillance and not research at Centers for Disease Control and Prevention, thus exempt from institutional review board review.

We assembled a retrospective cohort from surveillance data of pregnant women. The exposure of interest was prenatal exposure to HCV, and perinatal hepatitis C was the outcome. The Surveillance for Emerging Threats to Pregnant People and Infants Network conducts surveillance of pregnant women with HCV infection and their children (*7*). As of September 9, 2022, seven US jurisdictions (Georgia, Los Angeles County, Massachusetts, New York City, New York State, Pennsylvania, Tennessee) had contributed data on persons with HCV RNA detected during or within 1 year before pregnancy who had no evidence of treatment or clearance and who had live births during January 1, 2018–October 9, 2020. Children were determined to have perinatal hepatitis C if HCV RNA was detected or they had a reactive HCV antibody test during the recommended window (RNA at ≥2 months of age or antibody at ≥18 months of age) (*4*). Collection of data is ongoing to provide a complete picture of testing practices, including distinguishing those who were not tested from those who tested negative. We determined the expected number of children with perinatal hepatitis C by estimating 5.8% (95% CI 4.2%–7.8%) of live births exposed to HCV from included jurisdictions on the basis of a published estimate (*1*). 

A total of 2,266 children were born to pregnant women with hepatitis C during the surveillance period (Figure). Among those children, 408 (18%) were tested for HCV infection within the recommended window and 19 (1%) outside it. Forty-seven children (2%) had perinatal hepatitis C. Median age at initial positive test was 18.6 months. Perinatal HCV infection was detected at <18 months of age for 17 (36%) children and ≥18 months of age for 30 (64%) (Table). Of the 47 children with perinatal hepatitis C, 18 (38%) had a reactive HCV antibody test and HCV RNA detected on the same day, likely reflecting reflex laboratory testing. 

**Figure:**
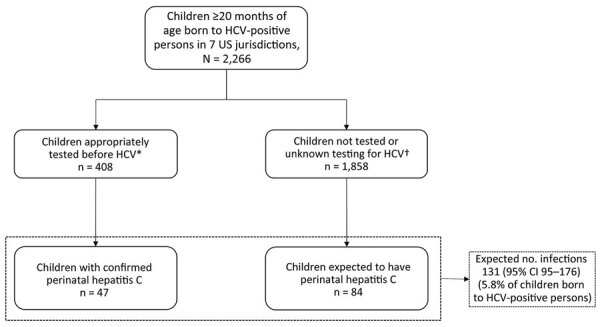
Observed and expected HCV infections among children with perinatal hepatitis C exposure in 7 US jurisdictions, 2018–2020. *Appropriate testing is considered test conducted at ≥2 months for HCV RNA or ≥18 months for HCV antibody. †May include children who tested negative for HCV, children whose tests were not reported to the health department, or children tested at an inappropriate age (<2 months for HCV RNA; <18 months for HCV antibody). HCV, Hepatitis C virus.

**Table:** Characteristics of 47 children with perinatal hepatitis C in a cohort of 2,266 children from 7 United States jurisdictions, by timing of test, 2018–2020

Characteristics	Children with perinatal hepatitis C	
First positive test <18 months of age,* n = 17 (36%)	First positive test ≥18 months of age,† n = 30 (64%)
Pregnant persons		
Age at HCV infection in pregnancy, y, median (IQR)	30 (24–33)	27 (23–32)
Race/ethnicity		
White, Non-Hispanic	15 (88)	27 (90)
Other	2 (12)	3 (10)
Education		
High school graduate and below	7 (41)	21 (70)
Some college and higher	6 (35)	9 (30)
Missing	4 (24)	0
Health insurance at delivery		
Public	13 (76)	28 (93)
Private/other/none	4 (24)	2 (7)
Substance use		
Any‡	10 (59)	22 (73)
None	2 (12)	2 (7)
Missing	5 (29)	6 (20)
Infants		
Neonatal abstinence syndrome§	4 (24)	12 (40)
Missing data	8 (47)	11 (37)
Neonatal intensive care unit admission	6 (35)	12 (40)
Missing data	1 (6)	0 (0)
Age at first positive test, mo, median (IQR)	7.3 (3.6–11.6)	19.4 (18.7–24.4)

*Values are no. (%) except as indicated. All tests were done by HCV RNA. HCV, hepatitis C virus. †Two infants first tested by positive HCV RNA at ≥18 mo; 28 infants first tested positive by HCV antibody at ≥18 mo. ‡Substance use includes alcohol, tobacco, cannabis, illicit use of opioids, and other illicit substances (eg, methamphetamines, cocaine). §Includes a diagnosis of neonatal abstinence syndrome or drug withdrawal syndrome in infant of dependent pregnant person (ICD-10 code P96.1) during the birth hospitalization.

The expected number (*1*) of children with perinatal hepatitis C by 20 months of age was 131 (95% CI 95–176), suggesting there were an additional 84 children with unidentified perinatal hepatitis C in this cohort. Therefore, only 36% (47/131) of children by 20 months of age who were expected to have perinatal hepatitis C within our cohort were reported to public health authorities. Potential reasons for this discrepancy include loss to follow-up (e.g., patients did not attend follow-up appointments), lack of awareness of the need for testing, delayed testing or testing too early, not completing ordered tests (*8*), or lack of reporting positive tests to health departments.

Limitations of this report include the fact that negative tests are not uniformly reportable across the jurisdictions we studied. However, medical record abstraction is ongoing to be able to describe testing practices, including those who were not tested or tested negative. In addition, the number of children included in this analysis may be underestimated if confirmatory testing occurred outside of the jurisdiction for the pregnant person or they were lost to follow-up before delivery. Last, although 5.8% is a pooled estimate for risk of vertical HCV infection, underlying differences between the prior study population and the population included in this analysis could affect risk (*1*).

This report identified more positive infants than a previous study (36% vs. 16%) (*6*), but both indicate that most children perinatally exposed to hepatitis C are not tested for infection. Understanding testing patterns among children with perinatal HCV exposure and current gaps in perinatal HCV testing and surveillance will help serve as a baseline for improving testing and surveillance to identify children with perinatal hepatitis C, connect them to the appropriate care, and move toward hepatitis C elimination.
